# Single-Domain Antibodies As Therapeutics against Human Viral Diseases

**DOI:** 10.3389/fimmu.2017.01802

**Published:** 2017-12-13

**Authors:** Yanling Wu, Shibo Jiang, Tianlei Ying

**Affiliations:** ^1^Key Laboratory of Medical Molecular Virology of Ministries of Education and Health, School of Basic Medical Sciences, Fudan University, Shanghai, China

**Keywords:** single-domain antibody, nanobody, viral disease, antiviral therapeutics, human immunodeficiency virus-1

## Abstract

In full-size formats, monoclonal antibodies have been highly successful as therapeutics against cancer and immune diseases. However, their large size leads to inaccessibility of some epitopes and relatively high production costs. As an alternative, single-domain antibodies (sdAbs) offer special advantages compared to full-size antibodies, including smaller size, larger number of accessible epitopes, relatively low production costs and improved robustness. Currently, sdAbs are being developed against a number of viruses, including human immunodeficiency virus-1 (HIV-1), influenza viruses, hepatitis C virus (HCV), respiratory syncytial virus (RSV), and enteric viruses. Although sdAbs are very potent inhibitors of viral infections, no sdAbs have been approved for clinical use against virial infection or any other diseases. In this review, we discuss the current state of research on sdAbs against viruses and their potential as therapeutics against human viral diseases.

## Introduction

Antibody-based therapeutics are enjoying significant clinical success, with over 70 such molecules approved by the US FDA and hundreds more in various phases of clinical trials (1). Notably, although antibodies have been proven to be effective against a number of diseases, most FDA-approved monoclonal antibodies (mAbs) are used to treat cancer and immune disorders (1, 2), and only one antiviral humanized mAb, palivizumab, has been approved as a prophylactic to prevent respiratory syncytial virus (RSV) infection in neonates and immunocompromised individuals (3). The development of therapeutic antibodies against viruses has been impeded by high production costs and limited commercial market. Moreover, the relatively large size of antibodies, which results in correspondingly low tissue accessibility and penetration, affects their therapeutic efficacy (4). Therefore, smaller-sized antibodies and engineered variants have become promising alternatives to full-size mAbs (5).

In 1989, researchers reported the isolation of stable mouse antibody VH domains that could bind antigens with relatively high affinity (20 nM), and the term “domain antibodies (dAbs)” was suggested (6). Moreover, in 1993, a unique class of “heavy-chain-only” antibodies (HCAbs) was found in the serum of camels. The variable domains of these HCAbs, referred to as VHHs, nanobodies (a term coined by Ablynx, a biopharmaceutical company) or single-domain antibodies (sdAbs) (7), represent the smallest naturally derived antigen-binding functional fragments (~15 kDa). These sdAbs maintain affinities and antigen-binding specificities comparable to those of full-size mAbs. Importantly, they are easy to engineer and more economical to produce; they also possess other unique and superior properties for a range of therapeutic applications.

Here, we review sdAbs in relation to their possible therapeutic applications against highly aggressive human viral diseases. Potential sdAb-based therapeutics against viruses that are particularly important for public health, such as human immunodeficiency virus-1 (HIV-1), influenza A virus, respiratory syncytial virus (RSV), hepatitis C virus (HCV), and enteric viruses are discussed (Table 1). We also provide insight into the current status of the sdAbs, their ongoing development, as well as future challenges toward their successful implementation for therapy of human viral diseases.

**Table 1:** Update of published sdAbs directed against viruses according to their binding sites [modified from Vanlandschoot et al. (8)].

Binding sites	Reference	Immunogen or panning antigen	Origin	Mechanism	Potency *in vitro*	Breadth	*In vivo*
**Extracellular targeting**							
HIV CD4-induced binding site (coreceptor binding site)	Chen et al. (9)	HIV-1 Envs	Phage-displayed human VH library	Neutralization	Fusion proteins with CD4 superior to bnAbs	Clade A, B, C, D	–

HIV coreceptor binding site	Matz et al. (10)	Trimeric gp140	Llama	Neutralization	IC_50_: 0.2–40 µg/ml	Subtypes A, C, G, and CRF01_AE, CRF02_AG	–

HIV CD4-binding site	McCoy et al. (11)	Trimeric HIV-1 gp140	Llama	Neutralization	IC_50_: 0.03–50 µg/ml	Subtypes A, B, C, D, G and CRF_01 AE, CRF_02AG, AC, ACD, BC, and CD	–

HIV-1 MPER	Gong et al. (12)	Gp41 MPER peptide	CH2 library	Neutralization	–	Clade B, C, D, E	–

HIV-1 MPER	Hulsik et al. (13)	Trimeric gp41	Llama	Neutralization	IC_50_ bivalent: clade A: 2.4–4.6 µg/ml; clade B: 0.2–33.4 µg/ml	Clade A and B	–

RSV F protein	Detalle et al. (14)	Recombinant F protein and inactivated RSV-A	Llama	Neutralization	IC_50_: subtype A: 0.1 nM; subtype B: 0.24 nM	RSV A and B subtypes	Reducing both nasal and lung RSV titers prophylactically or therapeutically

RSV prefusion F protein	Rossey et al. (15)	Prefusion conformation, DS-Cav1	Llama	Neutralization	IC_50_: subtype A: 0.038–0.089 nM; subtype B: 0.022–0.032 nM	RSV A and B subtypes	30 µg sdAbs administered intranasally prevent RSV replication in RSV-challenged mice

Influenza M2	Wei et al. (16)	M2 (H3N2)	Synthetic Camel VHH library	Neutralization	Minimal inhibitory concentration at 1.2 µM	H3N2 and H1N1	200 µg antibodies protect 60% mice with H1N1 virus challenge

Influenza HA	Ibanez et al. (17)	Recombinant H5N1-HA	Llama	Neutralization	–	H5N1	Prophylactic or therapeutic treatment to rescue mice against H5N1 challenge

Influenza HA	Tillib et al. (18)	Inactivated H5N2 virus	Camel	Neutralization	Minimal inhibitory concentration at 4.2 nM	H5N2	200 µg protect 100% mice against virus challenge

Influenza HA	Hufton et al. (19)	Recombinant H1-HA	Alpaca	Neutralization	IC_50_: 3.2–212.2 nM	H1N1	–

Influenza NA	Cardoso et al. (20)	N1rec	Alpaca	Neutralization	IC_50_ of monovalent: 425.2 and 374.9 nM; bivalent: 0.157 and 0.69 nM	Clade 1 and 2 H5N1	60 µg prophylactic treatment protect 100% mice against a lethal challenge with H5N1 and oseltamivir-resistant variant

Influenza NA	Harmsen et al. (21)	Mixtures of purified influenza viruses	Llama	–	–	All N subtypes	–

HCV E2	Tarr et al. (22)	E2 glycoprotein	Alpaca	Neutralization and cell-to-cell transmission	IC_50_: 1–10 µg/ml	Six major genotypes	–

HSV-2 glycoprotein D	Geoghegan et al. (23)	Recombinant gD2	Llama	Killing infected cells by conjugated immunotoxin	IC_50_ of 6.7 nM	HSV-2	–

Rotavirus	van der Vaart et al. (24)	Rhesus-monkey rotavirus serotype G3	Llama	Neutralization	IC_50_: <1 µg/ml	G3 rotavirus strain	Reduce the morbidity of rotavirus induced diarrhea in mice

Rotavirus VP6	Garaicoechea et al. (25); Vega et al. (26); Maffey et al. (27)	VP6 protein	Llama	Neutralization	IC_80_ of monovalent: 0.2–3.9 µg/ml; bivalent: >3.9 μg/ml	Group A Rotavirus	Monovalent VHH protects and treats against RVA-induced diarrhea in mice and gnotobiotic piglets

Norovirus P domain of VLP	Koromyslova and Hansman (28)	GII.10 VLP	Alpaca	Particle disassembly	–	GII.4, GII.10, and GII.12	–

Poliovirus receptor-binding site	Thys et al. (29); Schotte et al. (30); Strauss et al. (31)	Poliovirus type 1 Sabin strain	Dromedary	Neutralization	IC_50_: 0.007–0.69 µM; IC_90_: 0.017–1.77 µM	Poliovirus type I	–

**Intracellular targeting**							

HIV Vpr	Matz et al. (32)	Synthetic Vpr peptide	Llama	No inhibitory activity	–	–	–

Influenza virus nucleoprotein (NP)	Ashour et al. (33); Hanke et al. (34)	Influenza virus PR8	Alpaca	Block vRNP nuclear import, viral transcription, and replication	–	Common influenza virus strains	–

Influenza virus nucleoprotein (NP)	Schmidt et al. (35)	Inactivated IAV	Alpaca	Block IAV infection	–	Influenza A virus	–

HCV NS5B	Thueng-in et al. (36)	NS5BΔ55 of genotype 3a HCV	Humanized-camel phage library	Inhibition of RdRp catalytic activity	2–4 µg inhibit RdRp activity by 10–69% and 10 µg decrease HCV RNA inside the cells	HCV-JFH1	–

HCV NS3	Phalaphol et al. (37)	rNS3-C	Humanized-camel phage library	Inhibition of helicase activity	–	HCV-JFH1	–

HCV serine protease	Jittavisutthikul et al. (38)	rNS3/4A	Humanized-camel phage library	Inhibition of protease activity	–	HCV-JFH1	–

Ebola and Marburg nucleoprotein (NP)	Sherwood et al. (39, 40); Darling et al. (41)	*Ebolavirus* or MARV-Mus NP	Single-pot semisynthetic llama library	Inhibition of NP packaging	–	Genus specific	–

*–, Not determined*.

## Overview of Antiviral sdAbs

**Figure 1:**
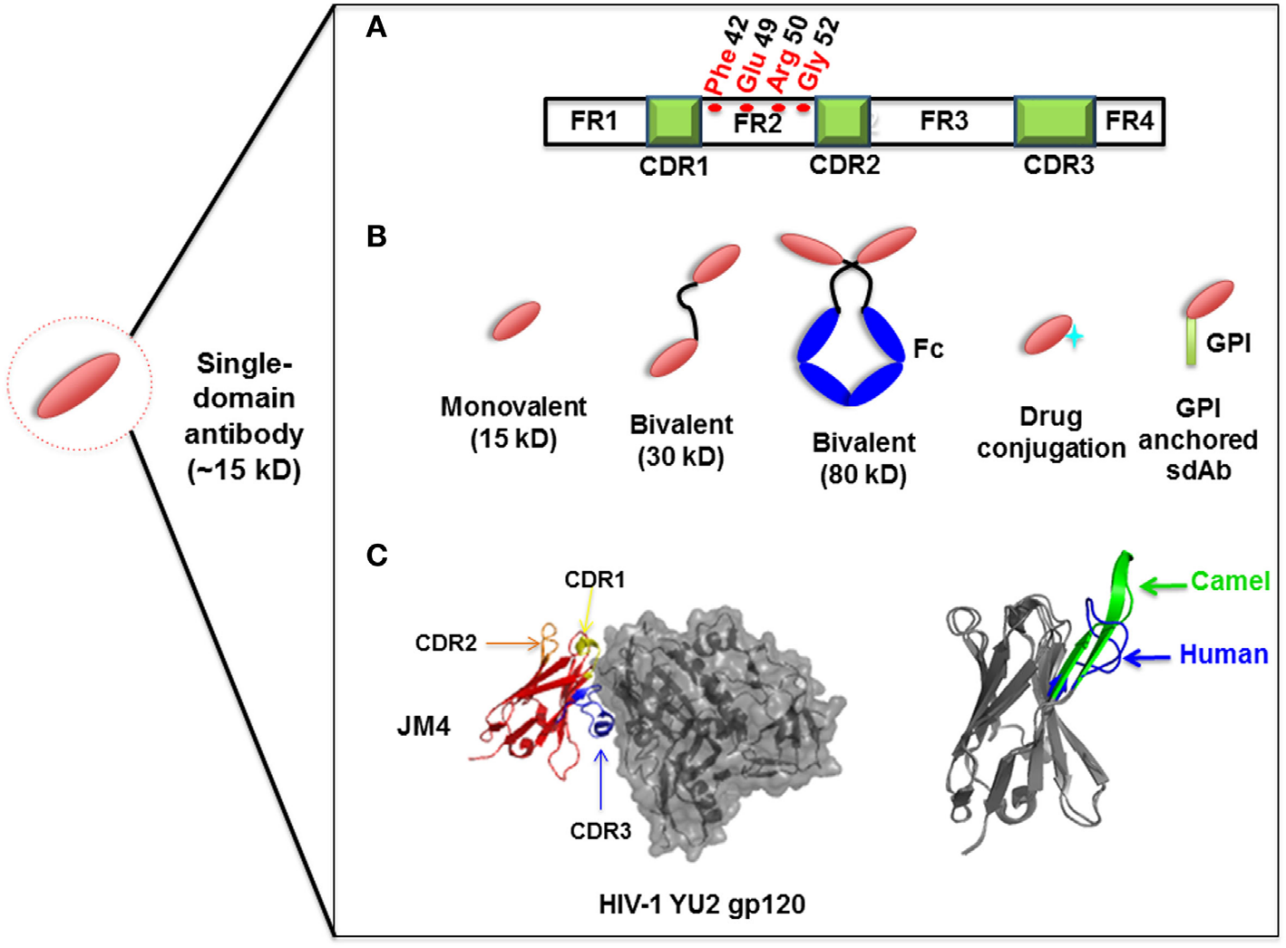
Representation of single-domain antibodies (sdAbs) and their characteristics. **(A)** Representation of camelid sdAb framework (FR) and complementarity-determining (CDR) regions, showing hydrophilic amino acids (Phe42, Glu49, Arg50, Gly52) in the FR2 region compared to conventional human VH (Val42, Gly49, Leu50, Trp52). **(B)** Schematic representation of sdAb-based engineered antibody constructs. **(C)** Neutralizing sdAb JM4 (PDB identifier 4LAJ) in complex with HIV-1 YU2 envelope gp120 glycoprotein, showing CDR1 (yellow), CDR2 (orange), and CDR3 (blue) and comparing CDR3 between human VH domain HEL4 (blue) (PDB identifier 1OHQ) and HIV-1 gp41 MPER-specific llama VHH 2H10 (green) (PDB identifier 4B50).

Compared to the conventional human antibody VH, a few crucial amino acids are substituted in the framework 2 region (FR2) and complementarity-determining regions (CDRs) of sdAbs. The highly conserved hydrophobic amino acids (Val47, Gly49, Leu50, Trp52) in FR2 region are replaced by hydrophilic amino acids (Phe42, Glu49, Arg50, Gly52) (Figure 1A) which are critical to the interaction of V_H_-V_L_, rendering the overall structure more hydrophilic and contributing to high stability, solubility and resistance to aggregation (42, 43). Moreover, sdAbs possess exceptional resistance to high temperatures and extreme pH (44), which makes them ideal candidates for developing viable treatment strategies against viruses in harsh environments such as the respiratory and gastrointestinal tracts. sdAbs can be easily administered *via* intranasal or oral route, directly to the site of viral infection (25, 45).

Owing to their increased hydrophilicity and single-polypeptide nature, sdAbs can be relatively efficiently produced in bacteria, yeast, mammalian cells or plant cells, enabling large-scale production at reasonable costs. Plant cell expression systems, such as transgenic crops can provide a particularly low-cost option. sdAbs expressed in such crops as rice do not require purification and can be stored at room temperature for a long period without compromising antiviral activity (46), which is beneficial in some areas where cold chains are difficult to maintain.

The small size of sdAbs (~15 kDa) also allows rapid tissue penetration, including the blood–brain barrier (47) and even neurospheres, in comparison to full-size mAbs (48), thus holding promise for therapy of neurotropic virus infections like rabies virus. Rabies virus is a model neurotropic virus, which can cause lethal brain infection in humans. Postexposure treatment with antirabies sdAbs can partly rescue mice from lethal disease and decrease the viral RNA load in the brain. In contrast, treatments with vaccines or human antirabies immune globulins could not meet this test, indicating that antirabies sdAbs can enter the brain and neutralize virus (49, 50). Still, because of their short half-life, sdAbs may not have enough time to cross the endothelial barriers in sufficient amounts to clear out virus, thus limiting the effect of sdAb treatment at the more advanced stages of infection.

Structural analysis of sdAbs in complex with their antigens revealed that some sdAbs display an extended CDR3. The convex conformations formed by the CDR3 of these sdAbs (Figure 1C) can target unique and cryptic epitopes and confer unique binding specificities by blocking the concave epitopes of antigens (31, 51).

**Figure 2:**
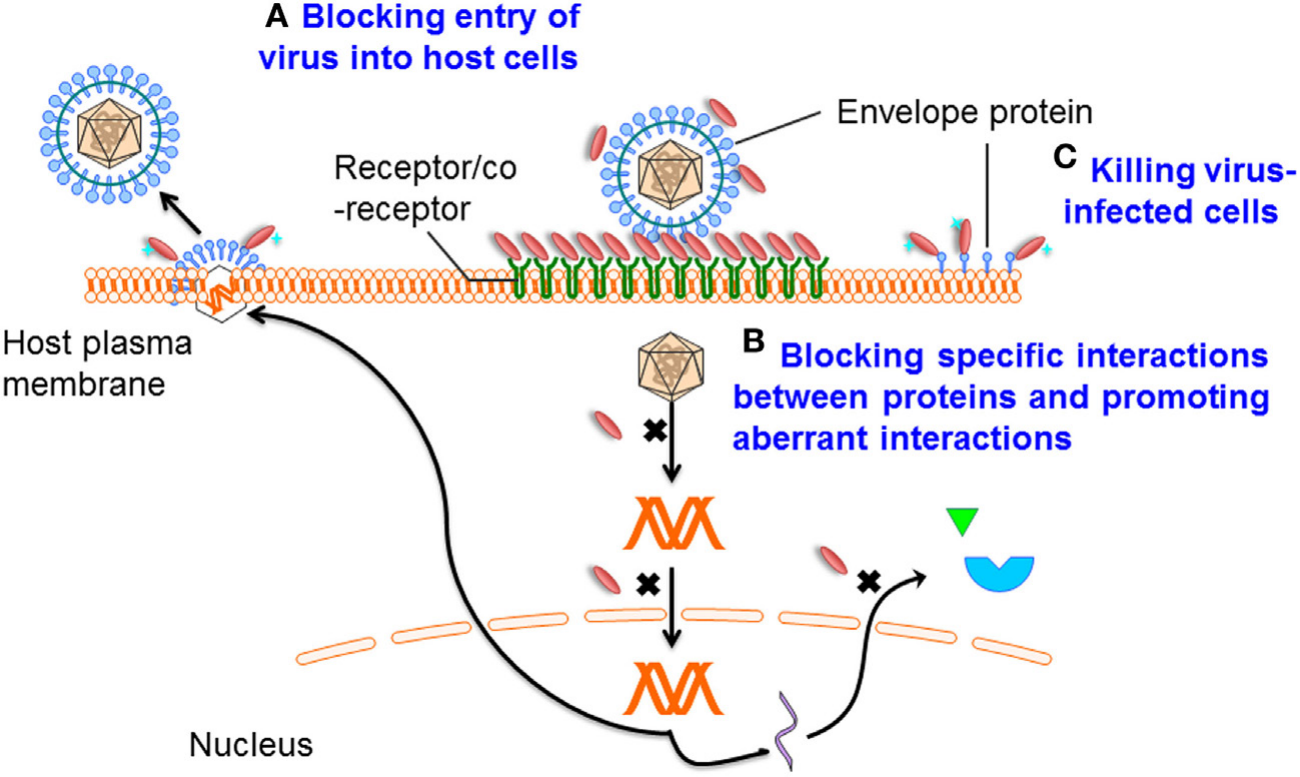
Mechanisms of single-domain antibody (sdAb)-based therapeutics against viruses. Mechanism A: preventing entry of the viral particle into host cells by targeting viral envelope proteins or receptors that mediate cell binding and membrane fusion; mechanism B: blocking specific interactions between virus/virus or virus/host proteins, promoting aberrant interactions, binding in the active sites of enzymes, or through recognition or stabilization of distinct conformations of their targets; and mechanism C: specifically killing virus-infected cells by drug-conjugated or toxin-fused sdAbs.

Single-domain antibodies can be easily engineered as multivalent constructs (Figure 1B). A number of studies indicated that multivalent formats are more effective than monovalent sdAbs in virus neutralization. For instance, it was found that a bivalent camelid VHH targeting H5N1 hemagglutinin was at least 60-fold more effective than the monovalent one in controlling virus replication (17, 20). Moreover, conversion of influenza hemagglutinin-specific and cross-neutralizing antibodies into a bivalent format can increase their breadth of subtype cross-reactive neutralization activity (19). ALX-0171, a trimeric RSV-neutralizing VHH that binds to an epitope similar to that of palivizumab, displayed more potent neutralization activity than palivizumab against prototypic RSV subtype A and B strains (14). Moreover, fusion with drugs, such as immunotoxins or cytotoxins, by site-specific conjugation to a C-terminal cysteine not only maintains the binding properties of sdAbs, but also increases their killing power against virus-infected cells (23) (Figure 2). Direct fusion to human serum albumin (HSA) (52) and PEGylation (53) can extend the serum half-life of sdAbs. However, such molecules have relatively large size that could lead to decreased inhibitory activity. Another attractive strategy for enhancing antibody pharmacokinetics by fusion to the Fc fragment of an IgG1 (54). Although these strategies increase the size of the antigen binders, the engineered molecules are still expected to target their epitopes more efficiently than full-length antibodies. A previous study reported improved half-life *in vivo* can be achieved by fusing sdAb with a small-sized HSA-binding peptide (15–20 kDa) and the resultant fusion protein showed the same neutralizing activity as that of unconjugated sdAb (9).

Currently, small-molecule drugs are widely available to treat infections caused by HIV-1 (55), HBV (56), HCV (57), as well as influenza viruses (58). For instance, since the discovery of HIV-1, more than 30 compounds have been approved for the treatment of HIV-1 infection (59). These drugs demonstrate that a number of cytosolic proteins could serve as ideal targets for inhibition of viral infections. Although the penetration of sdAbs through the cell membrane remains a problematic issue, many sdAbs have sufficient inherent stability to be functional in antigen-binding, even in the absence of the disulfide bond as occurring in the reducing environment of the cytoplasm. When expressed as intrabodies, they can bind antigens to inhibit viral replication (35, 41). Furthermore, the high binding specificity of sdAbs can only rarely be achieved by small-molecule drugs. Analysis of V-D-J gene rearrangement shows that camelid sdAbs share high similarity (greater than 80%) with the human IGHV3 gene family (44), indicating that the immunogenicity of camelid sdAbs could be low. Data from clinical trials of ALX-0171, an anti-RSV VHH, support this notion (45). These sdAbs can be “humanized” without significant loss of their specific activity (60). Moreover, a high-affinity and high-neutralization sdAb has been isolated from a large human VH-based phage display library (9). Therefore, camelid sdAbs and fully human sdAbs have potential as therapeutics against viral infections.

## Targets of Antiviral sdAbs

### Proteins Mediating Entry of Viruses into Host Cells

Infection is conditional on viral entry into host cells. Viral replication can then be initiated. This entry process is initially mediated by one or several viral proteins exposed on the virion surface and receptors or coreceptors on the host cell surface (61, 62). Proteins mediating viral entry are the promising targets for antibody-based antiviral therapy (Figure 2). Analysis of virus structure shows that many epitopes on the virion surface are hidden by deep invaginations or canyons and that they are, therefore, barely recognized by the large and typically flat antigen-binding sites of conventional antibodies (15, 63). More importantly, while many protruded epitopes on the viral surface are targeted by conventional antibodies, such epitopes can rapidly change conformation, resulting in the escape of virus from humoral immune responses (64). At the same time, these “hidden” epitopes, typically inaccessible to conventional antibodies, are well conserved across diverse viruses, making them good targets for sdAbs.

### sdAbs As Intrabodies Targeting Intracellular Proteins

In the virus life cycle, many proteins encoded by viral genomes play essential roles in replication. Therefore, interfering with the virus life cycle by interrupting the functions of these proteins is another effective strategy (Figure 2), as already demonstrated by their successful targeting by small-molecule inhibitors. However, antibodies do not pass the plasma membranes. By gene transfer, intrabodies expressed in the cytoplasm may have broad antiviral therapeutic applications. As a consequence of the reducing environment of the cytoplasm, the formation of disulfide bonds is prevented. For most conventional mAbs, or their fragments, such as Fab and scFv, correct folding and stability generally depend on the formation of intra-domain disulfide bonds. Indeed, it has been shown that antibody fragments expressed in the reducing environment are strongly destabilized (65). In contrast, sdAbs can be functionally expressed in the cytoplasm (35), suggesting that their activities or stabilities are less dependent on the disulfide bond formation. Furthermore, Darling et al. advanced an alternative approach to generate a dimeric intracellularly expressed sdAb against Filoviruses which targeted highly conserved C-terminal regions of nucleoprotein (NP). This dimeric sdAb can restrict viral packaging and inhibit Marburg and Ebola replication (41). As such, sdAbs represent a rich source of functional intrabodies of potential therapeutic importance.

## sdAbs as Potential Therapeutics Against Viruses

### Human Immunodeficiency Virus 1

The HIV-1 Env, a trimeric complex comprised of gp120 and gp41, typically serves as the main target for neutralizing antibodies. HIV-1 entry, the first step of the replication cycle, requires that gp120 engage the host cell surface CD4 and undergo a conformational change to bind either CCR5 or CXCR4, with subsequent fusion of cell and viral membrane mediated by gp41. Recently, a few highly potent and broadly neutralizing antibodies (bnAbs) have been identified from long-term HIV-infected individuals, including, for example, X5 (66), VRC01 (67), PGT121 (68), and 3BNC117 (69). Analysis of their binding models demonstrated that the key epitopes on gp120 for bnAbs are located at the CD4-binding site (CD4bs) and the coreceptor-binding site (CoRbs). The CD4bs is formed as a hydrophobic and recessed pocket (70, 71). This pocket is surrounded by five loop structures, which partially overlay the site and may prevent access to large antibodies (72). CoRbs epitopes are usually inaccessible until gp120 changes its conformation upon CD4 binding. Therefore, these antibodies that specifically bind to exposed epitopes following CD4-gp120 binding are also termed as CD4-induced (CD4i) antibodies, which, however, are also masked by flanking V2 and V3 loops (70). Moreover, the close proximity of viral and cellular membranes leaves only a very narrow space which may not be sufficient to accommodate conventional Ig antibodies (73). The membrane proximal external region (MPER) epitopes on gp41 may also be size-restricted (63). In fact, the transient fusion-intermediate conformation of gp41 (74) is only accessible after the conformational changes induced by receptor/coreceptor binding in Env. It is obvious that these steric constraints require smaller antibody fragments which have smaller paratopes to access these regions. Because of their small size, protruding CDR3 loops, and cleft-recognition properties, sdAbs can reach such inaccessible epitopes and block entry, in many cases more efficiently than the corresponding Fab and scFv fragments and the full-size antibodies.

The first human sdAb, m36, identified from a human antibody variable domain phage-displayed library, targets a highly conserved sterically restricted region on gp120 induced by CD4 binding. The neutralizing activity of m36 is, on average, higher than that of scFv m9 that is a matured derivative of X5 and superior to other known first-generation HIV-1 bnAbs (9). Larger-sized IgG variants of this antibody exhibited a significant reduction in overall neutralization potency compared to m36 (9). To date, m36 is the only reported HIV-1 inhibitor with exceptional potency and broad cross-reactivity based on human dAbs.

Using trimeric gp140 as immunogen in llamas and gp120 for selection, a panel of broadly neutralizing camelid sdAbs (VHHs) were identified that can bind to either CD4bs or CoRbs (10). These camelid sdAbs exhibited potent neutralizing activity against viruses expressing subtype B envelopes, including primary viral isolates resistant to some bnAbs, including 2G12, b12, 2F5, 4E10, PG9, and PG16 (10). One of the sdAbs, JM4, recognized a novel CD4i epitope including elements of both CD4bs and CoRbs (75). JM4 showed greater neutralization efficacy than known human CD4i IgGs as well as their Fab fragments, but IgG2b and IgG3 formats of JM4 showed dramatically enhanced breadth and potency (75). Further, Liu et al. developed GPI-anchored variable regions by genetically linking sdAbs with a glycosylphosphatidylinositol (GPI) attachment signal, which targeted lipid rafts of plasma membrane. Transduction of human CD4^+^ cell lines and primary CD4 T cells with GPI-VHH JM4 conferred broad and potent neutralization of HIV-1 and efficiently interfered with cell-cell transmission of HIV-1 and HIV-1 envelope-mediated fusion (76). One extremely potent and broad HIV-1-neutralizing sdAb from an immunized llama, J3, targeted the CD4-binding site and neutralized 96% of all strains tested (11). Notably, J3 exhibited high potency in blocking the cell–cell spread of HIV-1 from primary macrophages to CD4 T cells (77).

MPER-specific antibodies are among the broadest cross-reactive HIV-1 neutralizing antibodies (78). However, numerous studies have been performed with purified gp41 proteins and gp41-derived peptides in an attempt to induce such antibodies by immunization, but such attempts have, thus far, met with little success (79, 80). An MPER-specific VHH, 2H10, whose epitope (EQELLELDK) partially overlaps with that of 2F5, was elicited by immunizing llamas with gp41-MPER proteoliposomes (81). 2H10 bound to a linear epitope of gp41 with low nanomolar affinity. Analysis of its crystal structure revealed an extended CDR3 with a solvent-exposed tryptophan (W100) at its tip, which is required for its neutralizing activity (13). Increasing affinity by increasing avidity, as demonstrated for bivalent 2H10, led to the neutralization of various sensitive and resistant strains, including some Tier 2 viruses, with 100-fold higher potency than mAb 2F5. Although bivalent 2H10 lacks the potency and breadth of 2F5, 4E10, or 10E8, optimization of the immunization protocol, such as longer immunization schemes, may produce more extensive somatic mutations and yield antibodies with higher breadth and potency. Interestingly, an IgG1 CH2 domain-based dAb was generated from a phage library. This dAb binds to MPER and can neutralize a limited number of HIV-1 isolates (12). It also binds to FcRn. The term nanoantibody was coined for such CH2-based dAbs because nanoantibodies can mimic some of the functions of full-size antibodies (82).

Previous studies have already found that anti-Nef and anti-Rev intracellular sdAbs could efficiently block most of the activities of these viral proteins (83, 84) to inhibit HIV-1 replication. The HIV-1 viral regulatory protein (Vpr) is involved in regulation of efficient virus replication, and known to induce cell cycle arrest, apoptosis, and the enhancement of HIV-1 transcription in infected cells. Matz et al. isolated a panel of anti-Vpr sdAbs from two libraries of VHHs elicited by two immunized llamas with either a synthetic Vpr peptide or recombinant HIV-1 capsid protein (32). One of these VHHs was able to bind Vpr in the cytoplasm of eukaryotic cells, leading to its delocalization from the nucleus to the cytoplasm, but had no effect on the activities of Vpr (32). This problem may be solved by fusing sdAb with an SH3 domain, demonstrated by one study from the same research group which found that Neffins, composed of an anti-Nef sdAb and modified SH3 domain, inhibited all key activities of HIV-1 Nef (85).

### Influenza A Virus

Influenza viruses are currently classified into A, B, C, and D types on the basis of antigenic differences (86). In particular, highly pathogenic influenza A viruses occasionally cross the species barrier between domesticated birds and humans, such as the H5 and H7 subtypes, leading to seasonal epidemics and, sometimes, worldwide pandemics with high morbidity and mortality owing to severe and fatal acute respiratory diseases (87, 88). In the current decade, zoonotic outbreaks have posed significant threats to public health. However, currently available anti-influenza drugs are limited because of spontaneous virus mutations. Furthermore, such drugs often result in side effects and the emergence of drug-resistant viruses. Influenza A viruses are enveloped viruses antigenically consisting of two major membrane glycoproteins, hemagglutinin (HA), which mediates the binding of influenza virion to host cells and membrane fusion, and neuraminidase (NA), which is critical for the efficient release of newly synthesized influenza viruses by cleaving sialic acids from host cell receptor. While inhibitors specific for either the HA or NA glycoprotein (89, 90) can block virus infection, only HA glycoprotein mediates the virus entry process, making it a potential target for neutralizing antibodies. Several HA-targeting VHHs were isolated from llamas and were found to be specific for the H5N1 strain (17). One of these antibodies potently suppressed influenza A virus replication *in vivo* by intranasal administration. Bivalent antibodies showed 60-fold higher suppression than their monovalent counterparts and protected mice against a lethal challenge with H5N1 (17). In another study, Tillib et al. reported the development of potent camelid HA-specific sdAbs elicited by the immunization of a camel with inactivated avian influenza virus H5N2. The neutralizing activities of the original monovalent antiviral sdAbs are significantly enhanced both *in vitro* and *in vivo* by formatting procedure using the isoleucine zipper domain (ILZ) (18). The expression of ILZ formatted anti-HA sdAb *in vivo* for up to 14 days by an adenoviral vector resulted in prolonged protective effect against influenza virus (91). Decreased NA activities by blocking antibodies helped to protect a mouse model against H5N1 virus challenge (20).

Influenza M2 is a homotetrameric transmembrane protein that functions as a proton channel. M2 is required in several steps of influenza virus infection, including uncoating of the viral ribonucleoprotein core in endosomes, viral assembly and release (92, 93). In contrast to HA and NA, the N-terminal extracellular domain of M2 (M2e) has 24 residues remarkably conserved in all human influenza A strains. As such, neutralizing antibodies against M2e are thought to offer broad protection. Indeed, M2-7A, an sdAb that specifically bound M2, showed broadly cross-reactive neutralization for both amantadine-sensitive and -resistant viruses *in vitro* and protected mice from lethal influenza virus challenge (16).

Given the continued relevance of influenza virus as a serious health threat and its ability to rapidly acquire resistance against drugs or escape from immune responses by antigenic drift, the less variable influenza virus proteins, including virus NP, may prove to be alternative targets for intervention. A panel of sdAbs was generated against NP, a viral protein essential for nuclear trafficking and packaging of the influenza virus genome (33, 34). These sdAbs disrupt virus replication by preventing nuclear import of viral ribonucleoproteins (vRNPs). One such sdAb, termed as αNP-VHH1, exhibited antiviral activity similar to that of Mx protein. Analysis of the crystal structure of this VHH in complex with NP revealed that the binding site overlaps regions associated with viral sensitivity to Mx proteins and is not conserved on the body domain of NP implicated in interactions with host factors (34). Schmidt et al. developed a highly efficient screening approach to identify antiviral sdAbs that confer a phenotype to cells when expressed intracellularly. Anti-NP sdAbs that specifically interact with their respective nucleoproteins protect human cells from lethal Influenza A virus infection by preventing nuclear import of viral vRNPs (35). So far, more than 20 NP-specific sdAbs have been characterized as binding to at least four unique binding sites on NP (33, 35). Continued efforts in this direction might help to map more precisely the contributions of different NP surfaces to the influenza virus life cycle and inspire the development of novel antivirals.

### Respiratory Syncytial Virus

Respiratory syncytial virus infection causes serious or even fatal lower respiratory tract infections in infants. It is estimated that about 3.4 million infants are infected by RSV annually and more than 3 million of them develop severe bronchiolitis or pneumonia (94). Currently, neither licensed RSV vaccines nor specific anti-RSV therapeutics are available. RSV has two classes of transmembrane glycoproteins on the viral surface, fusion (F) protein, and attachment (G) protein. Receptor binding is mediated by G protein, followed by fusion of viral and cell membrane and viral entry facilitated by F protein (95). Therefore, both proteins contain epitopes for neutralizing antibodies. The F protein shares high similarity between RSV subgroups A and B (89% amino acid identity) and lower glycosylation compared to G protein, and is thus considered an ideal target for developing anti-RSV agents. The humanized mAb palivizumab (Synagis) binds to RSV F protein and neutralizes RSV by preventing fusion of the viral and host cell membrane (96). Although reducing hospitalizations when administered prophylactically, its high cost and limited efficacy have restricted its use to high-risk infants (97). Recently, an RSV-neutralizing, F protein-specific sdAb, Nb017, was identified from immune libraries of llamas (14). Fusion of three monovalent Nb017s linked by two GS linkers formed a trimeric antibody, ALX-0171, which binds antigenic site II epitope on RSV F, similar to that of palivizumab. ALX-0171 showed exceptional cross-reactive neutralization *in vitro*, much higher than that of palivizumab. Furthermore, ALX-0171, when directly delivered prophylactically or therapeutically to the sites of infection, was shown to be highly effective in reducing RSV replication in both nasal passage and lung (14). In a phase I/IIa trial, the viral loads in nasal swabs of hospitalized RSV-infected children were reduced by daily treatment for three consecutive days with ALX-0171 delivered by an inhalation device (45). Several studies reported that antibodies specifically binding to prefusion conformation of F protein exhibited more robust neutralizing activity than conformation-independent antibodies (98–100). Two llama-derived, prefusion F-specific sdAbs were identified to have neutralizing activity against RSV A and B subtypes superior to that of mAb palivizumab and motavizumab (15). Crystallization studies revealed that both sdAbs bind to a conserved cavity epitope formed by two F protomers, illustrating that the sdAbs preferentially bind to clefts or cavities. Prophylactic treatments with 30 µg sdAbs administered intranasally prevented RSV replication in RSV-challenged mice (15). Such sdAbs with extraordinarily high RSV-neutralizing activity could be developed as therapeutics for the treatment of RSV infections.

### Hepatitis C Virus

Hepatitis C virus infection typically manifests as chronic hepatitis which often progresses to fatal cirrhosis and hepatocellular carcinoma. At present, no effective preventive treatment is available, and currently approved therapeutics are limited by relatively high cost (101). HCV envelope glycoprotein E2 is well conserved across all genotypes and can bind to host cell receptors, including CD81 and scavenger receptor class B type I (SR-BI), making E2 an attractive target for neutralizing antibodies (102). HCV E2-specific neutralizing antibodies have been shown to neutralize genetically diverse HCV isolates and effectively prevent and treat HCV infection in a human liver-chimeric mouse model (103) and chimpanzees (104). More recently, treatment of a neutralizing human mAb targeting HCV E2 significantly delayed viral rebound in patients infected with HCV 1a following liver transplantation (105). From a phage-library displaying an sdAb repertoire of alpaca immunized with HCV E2 glycoprotein, one E2-specific and potent cross-reactive neutralizing sdAb, D03, was identified. D03 recognizes a novel conserved epitope overlapping with that of the CD81 binding site. Structural analysis of D03 revealed a long CDR3 (20 residues) folding over part of the framework. Between the upstream part of CDR2 and CDR3, a disulfide bridge is formed that serves to restrict the flexibility of CDR3, thereby allowing maximal accessibility to the tip region of D03. D03 displayed potently neutralizing activity against a panel of HCV pseudoparticles representing six genotypes by interfering with E2–CD81 interaction, with an IC_50_ ranging between 1 and 10 µg/ml for most isolates. In addition, D03 efficiently inhibited cell-to-cell transmission of HCV, which is resistant to CD81 binding-site bnAbs (22). Although several sdAbs have entered clinical trials, this antibody is the first one that can prevent both cell-free and direct cell-to-cell transmission of a virus, highlighting its potential for the development as a clinically useful entry inhibitor.

A number of anti-NS5B, anti-NS3, and antiprotease sdAbs were identified from a humanized-camel naive VHH phage-display library (36–38). The three proteins are non-structural (NS) proteins, but with different enzymatic activities, and they are involved in HCV replication. By linking of these sdAbs to a cell-penetrating peptide, penetratin, these cell-penetrable sdAbs could suppress the heterologous HCV replication at different degrees, as shown by reduction of both intracellular and released viral RNA copies. Their recognized epitopes are located in the catalytic grooves of the enzymes. Therefore, a cocktail of sdAbs specific to multiple epitopes of HCV highly conserved regions and viral enzymes or enzymatically active proteins may be safe, broadly effective, and relatively mutation-tolerant anti-HCV agents.

### Herpes Simplex Virus 2

Herpes simplex virus 2 is one of the most prevalent sexually transmitted viruses (106). HSV-2 infection is often described as a relatively mild and generally not life-threatening disease, but it is still one that significantly contributes to the acquisition of HIV-1 infection (107). Despite decades of intense research dedicated to preventing the sexual transmission of HSV-2, no broadly protective vaccine or therapeutic is available. Glycoprotein D (gD) mainly mediates membrane fusion by the interaction with its receptors, which serves as a key step for successful virus entry (108), as demonstrated by the finding that neutralizing antibodies targeting gD displayed effective protection against HSV infection (109). One gD-specific sdAb, R33, was identified from a VHH immune library obtained after immunizing a llama with HSV-2 gD, but did not show neutralization against HSV-2 virus *in vitro*. Fusion of the cytotoxic domain of *Pseudomonas aeruginosa* exotoxin A (R33ExoA) to R33 did display specific and potent killing of HSV-2-infected cells, with an IC_50_ of 6.7 nM, indicating that R33 can deliver toxins or drugs to HSV-2-infected cells to reduce their nonspecific toxicity to normal cells and diminish side effects (23). This study confirmed that the antiviral activity of sdAbs could be increased by fusing them to toxins to generate immunotoxins.

### Enteric Viruses

Up to now, gastroenteritis remains a critical health issue worldwide. Rotaviruses (RVs) and noroviruses (NoVs) are the two primary viral etiologies responsible for nonbacterial diarrhea in adults or children. Although the currently licensed rotavirus vaccines are efficacious in preventing rotavirus infection, no drugs have been approved for norovirus treatment. An antirotavirus VHH [termed antirotavirus protein (ARP1)] from a llama immunized with rhesus-monkey group A rotavirus (RVA) strain has been shown to neutralize 11 strains and displayed preventive and therapeutic potency against RVA-induced diarrhea in a neonatal mouse model (24, 46, 110, 111). Interestingly, mitigation of the symptoms was also achieved with freeze-dried ARP1, allowing it to be applied in areas where cold chains are difficult to maintain. Moreover, Pant et al. developed secreted or anchored antirotavirus VHH expressed in *Lactobacillus paracasei* to directly deliver therapeutic agents for the treatment of gastroenteritis (110, 111). Currently, the first clinical trial was performed in infants with RVA-associated diarrhea. This resulted in significant reduction in the frequency of stool output and no side effects after oral ARP1 therapy (112). Another VHH, 3B2, which is specific to the inner capsid protein VP6, showed more broadly neutralizing capacity against RVA strains at lower doses (25). 3B2 conferred protection against RVA-induced diarrhea not only in neonatal mice (25), but also in gnotobiotic piglets (26), an animal model that has been widely used to study human RVA pathogenesis. Furthermore, postinfection therapeutic treatment with 3B2 or 2KD1 in a neonatal mouse model significantly reduced duration of RVA-induced diarrhea (27).

Human NoVs are single-stranded RNA viruses whose capsid protein is composed of shell (S) and protruding (P) domains linked by a flexible hinge region (113). Because of the inability to cultivate NoVs in cells and continually evolving strains, no vaccines or therapeutics against NoVs are available. The lower region on the P domain contains several highly conserved residues among the genetically distinct genogroup II (GII) noroviruses, which is a dominant epitope recognized by broadly reactive mAbs (114, 115). Nano-85 and Nano-25 were isolated from alpha immunized with GII norovirus virus-like particles. They both bind to the region of P domain that forms occluded sites on the particles, as demonstrated by cryoelectron microscopy structures of sdAbs with viral particle complexes (28). Different from strain-dependent Nano-25, Nano-85 was broadly reactive to several different GII genotypes, including GII.4, GII.10, and GII.12. Furthermore, the binding of Nano-85 to intact particles caused the particles to disassemble *in vitro* (28), but the underlying mechanism needs to be further elucidated.

## Conclusion and Perspectives

In many cases, no vaccines or effective therapeutics are available to combat pathogenic viruses. As an alternative to full-size antibody-based antiviral therapeutics, sdAbs have begun to attract considerable scientific attention. sdAbs are characterized by their unique biophysical and pharmacological features, such as small size, high stability, excellent expression yield in prokaryotic and eukaryotic systems, and low immunogenicity. Antiviral sdAbs with nanomolar neutralization potency could be directly isolated from elicited sdAb repertoires of immunized animals when displayed on the surface of phage, yeast (116) or mammalian cells (35), or from large naive sdAb libraries (9). Moreover, sdAbs serve as excellent molecules for making multivalent constructs. Multimerization of sdAbs can easily increase affinity and potency. Fusion of several sdAbs that recognize different epitopes together might reduce the emergence of resistant virus during treatment. In this respect, sdAbs have an advantage over IgG, as the production of IgG-based bispecific antibodies involves the specific combination of four different polypeptides. Such novel multivalent constructs could be very effective *in vivo*. Finally, as discussed above, future research might focus on exploiting the ability of sdAbs, as intrabodies, to target intracellular proteins.

Despite these advantages, two major issues need to be addressed before sdAbs can be considered for *in vivo* use: their short half-life in circulation and possible immunogenicity. Their relatively short half-life *in vivo* could be improved by various strategies, most of which may, however, lead to increased size and decreased inhibitory activity. For instance, one of the most efficient strategies in extending half-life is to fuse sdAbs with human IgG1 Fc. IgG1 Fc can bind to the neonatal Fc receptor (FcRn) and rescue the fused sdAbs from endosomal degradation, but such fusion will result in greatly increasing the size of sdAbs from ~15 to ~90 kDa. This could be partially addressed by the replacement of wild-type dimeric IgG1 Fc with some novel engineered Fc-based constructs. For example, we previously developed monomeric IgG1 CH3 or CH2–CH3 hybrids which have relatively small size (~15 kDa) and FcRn-binding capability (117–120). Such fusion proteins were easy to produce, stable, and retained the antigen-binding capability of the sdAbs (117, 121).

To reduce the risk of immunogenicity, strategies for the humanization of camelid-derived sdAbs have also become available in recent years. However, these strategies have some limitations, including the loss of heat refoldability upon humanization and the requirement to maintain two camelid residues within the FR2 (60). For biomedical applications, human VH sdAbs would be preferable because of the limited immunogenicity in patients. Although isolated human VH single domains generally display poor stability that causes aggregation, the poor biophysical properties of human VH single domains have recently been improved by the identification and introduction of mutations that allow maximal transfer of the hydrophobic to hydrophilic V_H_/V_L_ interface and do so by direct selection from phage libraries (122, 123). The grafting of CDRs from naive repertoire or immunized individuals into such human sdAb scaffolds could result in the development of fully human sdAbs against various targets. With advancing antibody-related technologies, we can anticipate the development of humanized or fully human sdAbs with minimal immunogenicity as commercially attractive therapeutics to fight viral diseases.

## Author Contributions

YW, SJ, and TY conceived and wrote the article.

## Conflict of Interest Statement

The authors declare that the article content was composed in the absence of any commercial or financial relationships that could be construed as a potential conflict of interest.

## References

[1] Reichert JM Antibodies to watch in 2017. mAbs (2017) 9(2):167–81. 10.1080/19420862.2016.1269580 27960628 PMC5297518

[2] Weiner GJ. Building better monoclonal antibody-based therapeutics. Nat Rev Cancer (2015) 15(6):361–70. 10.1038/nrc3930 25998715 PMC4491443

[3] Scott LJ Lamb HM. Palivizumab. Drugs (1999) 58(2):305–11; discussion 312–3. 10.2165/00003495-199958020-00009 10473022

[4] Samaranayake H Wirth T Schenkwein D Räty JK Ylä-Herttuala S. Challenges in monoclonal antibody-based therapies. Ann Med (2009) 41(5):322–31. 10.1080/07853890802698842 19234897

[5] Holliger P Hudson PJ Engineered antibody fragments and the rise of single domains. Nat Biotech (2005) 23(9):1126–36. 10.1038/nbt1142 16151406

[6] Ward ES Gussow D Griffiths AD Jones PT Winter G. Binding activities of a repertoire of single immunoglobulin variable domains secreted from Escherichia coli. Nature (1989) 341(6242):544–6. 10.1038/341544a0 2677748

[7] Hamers-Casterman C Atarhouch T Muyldermans S. Naturally occurring antibodies devoid of light chains. Nature (1993) 363(6428):446–8. 10.1038/363446a0 8502296

[8] Vanlandschoot P Stortelers C Beirnaert E Ibañez LI Schepens B Depla E Nanobodies^®^: new ammunition to battle viruses. Antiviral Res (2011) 92(3):389–407. 10.1016/j.antiviral.2011.09.002 21939690

[9] Chen W Zhu Z Feng Y Dimitrov DS. Human domain antibodies to conserved sterically restricted regions on gp120 as exceptionally potent cross-reactive HIV-1 neutralizers. Proc Natl Acad Sci U S A (2008) 105(44):17121–6. 10.1073/pnas.0805297105 18957538 PMC2579388

[10] Matz J Kessler P Bouchet J Combes O Ramos OH Barin F Straightforward selection of broadly neutralizing single-domain antibodies targeting the conserved CD4 and coreceptor binding sites of HIV-1 gp120. J Virol (2013) 87(2):1137–49. 10.1128/JVI.00461-12 23152508 PMC3554102

[11] McCoy LE Quigley AF Strokappe NM Bulmer-Thomas B Seaman MS Mortier D Potent and broad neutralization of HIV-1 by a llama antibody elicited by immunization. J Exp Med (2012) 209(6):1091–103. 10.1084/jem.20112655 22641382 PMC3371729

[12] Gong R Wang Y Ying T Dimitrov DS. Bispecific engineered antibody domains (nanoantibodies) that interact noncompetitively with an HIV-1 neutralizing epitope and FcRn. PLoS One (2012) 7(8):e42288. 10.1371/journal.pone.0042288 22879932 PMC3413693

[13] Lutje Hulsik D Liu YY Strokappe NM Battella S El Khattabi M McCoy LE A gp41 MPER-specific llama VHH requires a hydrophobic CDR3 for neutralization but not for antigen recognition. PLoS Pathog (2013) 9(3):e1003202. 10.1371/journal.ppat.1003202 23505368 PMC3591319

[14] Detalle L Stohr T Palomo C Piedra PA Gilbert BE Mas V Generation and characterization of ALX-0171, a potent novel therapeutic nanobody for the treatment of respiratory syncytial virus infection. Antimicrob Agents Chemother (2015) 60(1):6–13. 10.1128/AAC.01802-15 26438495 PMC4704182

[15] Rossey I Gilman MS Kabeche SC Sedeyn K Wrapp D Kanekiyo M Potent single-domain antibodies that arrest respiratory syncytial virus fusion protein in its prefusion state. Nat Commun (2017) 8:14158 10.1038/ncomms14158 28194013 PMC5316805

[16] Wei G Meng W Guo H Pan W Liu J Peng T Potent neutralization of influenza A virus by a single-domain antibody blocking M2 ion channel protein. PLoS One (2011) 6(12):e28309. 10.1371/journal.pone.0028309 22164266 PMC3229572

[17] Ibanez LI De Filette M Hultberg A Verrips T Temperton N Weiss RA Nanobodies with in vitro neutralizing activity protect mice against H5N1 influenza virus infection. J Infect Dis (2011) 203(8):1063–72. 10.1093/infdis/jiq168 21450996

[18] Tillib SV Ivanova TI Vasilev LA Rutovskaya MV Saakyan SA Gribova IY Formatted single-domain antibodies can protect mice against infection with influenza virus (H5N2). Antiviral Res (2013) 97(3):245–54. 10.1016/j.antiviral.2012.12.014 23274623

[19] Hufton SE Risley P Ball CR Major D Engelhardt OG Poole S. The breadth of cross sub-type neutralisation activity of a single domain antibody to influenza hemagglutinin can be increased by antibody valency. PLoS One (2014) 9(8):e103294. 10.1371/journal.pone.0103294 25084445 PMC4118869

[20] Cardoso FM Ibanez LI Van den Hoecke S De Baets S Smet A Roose K Single-domain antibodies targeting neuraminidase protect against an H5N1 influenza virus challenge. J Virol (2014) 88(15):8278–96. 10.1128/JVI.03178-13 24829341 PMC4135927

[21] Harmsen M Blokker J Pritz-Verschuren S Bartelink W van der Burg H Koch G Isolation of panels of llama single-domain antibody fragments binding all nine neuraminidase subtypes of influenza A virus. Antibodies (2013) 2(2):168–92. 10.3390/antib2020168

[22] Tarr AW Lafaye P Meredith L Damier-Piolle L Urbanowicz RA Meola A An alpaca nanobody inhibits hepatitis C virus entry and cell-to-cell transmission. Hepatology (2013) 58(3):932–9. 10.1002/hep.26430 23553604

[23] Geoghegan EM Zhang H Desai PJ Biragyn A Markham RB. Antiviral activity of a single-domain antibody immunotoxin binding to glycoprotein D of herpes simplex virus 2. Antimicrob Agents Chemother (2015) 59(1):527–35. 10.1128/AAC.03818-14 25385102 PMC4291438

[24] van der Vaart JM Pant N Wolvers D Bezemer S Hermans PW Bellamy K Reduction in morbidity of rotavirus induced diarrhoea in mice by yeast produced monovalent llama-derived antibody fragments. Vaccine (2006) 24(19):4130–7. 10.1016/j.vaccine.2006.02.045 16616802

[25] Garaicoechea L Olichon A Marcoppido G Wigdorovitz A Mozgovoj M Saif L Llama-derived single-chain antibody fragments directed to rotavirus VP6 protein possess broad neutralizing activity in vitro and confer protection against diarrhea in mice. J Virol (2008) 82(19):9753–64. 10.1128/JVI.00436-08 18632867 PMC2546978

[26] Vega CG Bok M Vlasova AN Chattha KS Gomez-Sebastian S Nunez C Recombinant monovalent llama-derived antibody fragments (VHH) to rotavirus VP6 protect neonatal gnotobiotic piglets against human rotavirus-induced diarrhea. PLoS Pathog (2013) 9(5):e1003334. 10.1371/journal.ppat.1003334 23658521 PMC3642062

[27] Maffey L Vega CG Miño S Garaicoechea L Parreño V. Anti-VP6 VHH: an experimental treatment for rotavirus A-associated disease. PLoS One (2016) 11(9):e0162351. 10.1371/journal.pone.0162351 27603013 PMC5014449

[28] Koromyslova AD Hansman GS. Nanobody binding to a conserved epitope promotes norovirus particle disassembly. J Virol (2015) 89(5):2718–30. 10.1128/JVI.03176-14 25520510 PMC4325747

[29] Thys B Schotte L Muyldermans S Wernery U Hassanzadeh-Ghassabeh G Rombaut B. In vitro antiviral activity of single domain antibody fragments against poliovirus. Antiviral Res (2010) 87(2):257–64. 10.1016/j.antiviral.2010.05.012 20566349

[30] Schotte L Strauss M Thys B Halewyck H Filman DJ Bostina M Mechanism of action and capsid-stabilizing properties of VHHs with an in vitro antipolioviral activity. J Virol (2014) 88(8):4403–13. 10.1128/JVI.03402-13 24501405 PMC3993733

[31] Strauss M Schotte L Thys B Filman DJ Hogle JM. Five of five VHHs neutralizing poliovirus bind the receptor-binding site. J Virol (2016) 90(7):3496–505. 10.1128/JVI.03017-15 26764003 PMC4794687

[32] Matz J Herate C Bouchet J Dusetti N Gayet O Baty D Selection of intracellular single-domain antibodies targeting the HIV-1 Vpr protein by cytoplasmic yeast two-hybrid system. PLoS One (2014) 9(12):e113729. 10.1371/journal.pone.0113729 25436999 PMC4249982

[33] Ashour J Schmidt FI Hanke L Cragnolini J Cavallari M Altenburg A Intracellular expression of camelid single-domain antibodies specific for influenza virus nucleoprotein uncovers distinct features of its nuclear localization. J Virol (2015) 89(5):2792–800. 10.1128/JVI.02693-14 25540369 PMC4325724

[34] Hanke L Knockenhauer KE Brewer RC van Diest E Schmidt FI Schwartz TU The antiviral mechanism of an influenza A virus nucleoprotein-specific single-domain antibody fragment. MBio (2016) 7(6):e01569–16. 10.1128/mBio.01569-16 27965447 PMC5156300

[35] Schmidt FI Hanke L Morin B Brewer R Brusic V Whelan SP Phenotypic lentivirus screens to identify functional single domain antibodies. Nat Microbiol (2016) 1(8):16080. 10.1038/nmicrobiol.2016.80 27573105 PMC5010022

[36] Thueng-in K Thanongsaksrikul J Srimanote P Bangphoomi K Poungpair O Maneewatch S Cell penetrable humanized-VH/V(H)H that inhibit RNA dependent RNA polymerase (NS5B) of HCV. PLoS One (2012) 7(11):e49254. 10.1371/journal.pone.0049254 23145135 PMC3493538

[37] Phalaphol A Thueng-In K Thanongsaksrikul J Poungpair O Bangphoomi K Sookrung N Humanized-VH/VHH that inhibit HCV replication by interfering with the virus helicase activity. J Virol Methods (2013) 194(1–2):289–99. 10.1016/j.jviromet.2013.08.032 24036073

[38] Jittavisutthikul S Thanongsaksrikul J Thueng-In K Chulanetra M Srimanote P Seesuay W Humanized-VHH transbodies that inhibit HCV protease and replication. Viruses (2015) 7(4):2030–56. 10.3390/v7042030 25903832 PMC4411689

[39] Sherwood LJ Osborn LE Carrion R Patterson JL Hayhurst A. Rapid assembly of sensitive antigen-capture assays for Marburg virus, using in vitro selection of llama single-domain antibodies, at biosafety level 4. J Infect Dis (2007) 196:S213–9. 10.1086/520586 17940952

[40] Sherwood LJ Hayhurst A. Ebolavirus nucleoprotein C-termini potently attract single domain antibodies enabling monoclonal affinity reagent sandwich assay (MARSA) formulation. PLoS One (2013) 8(4):e61232. 10.1371/journal.pone.0061232 23577211 PMC3618483

[41] Darling TL Sherwood LJ Hayhurst A. Intracellular crosslinking of filoviral nucleoproteins with xintrabodies restricts viral packaging. Front Immunol (2017) 8:1197. 10.3389/fimmu.2017.01197 29021793 PMC5623874

[42] Muyldermans S Atarhouch T Saldanha J Barbosa JARG Hamers R Sequence and structure of V-H domain from naturally-occurring camel heavy-chain immunoglobulins lacking light-chains. Protein Eng (1994) 7(9):1129–35. 10.1093/Protein/7.9.1129 7831284

[43] Vu KB Ghahroudi MA Wyns L Muyldermans S Comparison of llama V-H sequences from conventional and heavy chain antibodies. Mol Immunol (1997) 34(16–17):1121–31. 10.1016/S0161-5890(97)00146-6 9566760

[44] Muyldermans S. Nanobodies: natural single-domain antibodies. Annu Rev Biochem (2013) 82:775–97. 10.1146/annurev-biochem-063011-092449 23495938

[45] Van Heeke G Allosery K De Brabandere V De Smedt T Detalle L de Fougerolles A. Nanobodies^®^ as inhaled biotherapeutics for lung diseases. Pharmacol Ther (2017) 169:47–56. 10.1016/j.pharmthera.2016.06.012 27373507

[46] Tokuhara D Alvarez B Mejima M Hiroiwa T Takahashi Y Kurokawa S Rice-based oral antibody fragment prophylaxis and therapy against rotavirus infection. J Clin Invest (2013) 123(9):3829–38. 10.1172/JCI70266 23925294 PMC3754275

[47] Rutgers KS Nabuurs RJA van den Berg SAA Schenk GJ Rotman M Verrips CT Transmigration of beta amyloid specific heavy chain antibody fragments across the in vitro blood–brain barrier. Neuroscience (2011) 190:37–42. 10.1016/j.neuroscience.2011.05.076 21683126

[48] Rotman M Welling MM Bunschoten A de Backer ME Rip J Nabuurs RJA Enhanced glutathione PEGylated liposomal brain delivery of an anti-amyloid single domain antibody fragment in a mouse model for Alzheimer’s disease. J Control Release (2015) 203:40–50. 10.1016/j.jconrel.2015.02.012 25668771

[49] Terryn S Francart A Lamoral S Hultberg A Rommelaere H Wittelsberger A Protective effect of different anti-rabies virus VHH constructs against rabies disease in mice. PLoS One (2014) 9(10):e109367. 10.1371/journal.pone.0109367 25347556 PMC4210127

[50] Terryn S Francart A Rommelaere H Stortelers C Van Gucht S. Post-exposure treatment with anti-rabies VHH and vaccine significantly improves protection of mice from lethal rabies infection. PLoS Negl Trop Dis (2016) 10(8):e0004902. 10.1371/journal.pntd.0004902 27483431 PMC4970669

[51] Garza JA Taylor AB Sherwood LJ Hart PJ Hayhurst A Unveiling a drift resistant cryptotope within marburgvirus nucleoprotein recognized by llama single-domain antibodies. Front Immunol (2017) 8:1234 10.3389/fimmu.2017.01234 29038656 PMC5630700

[52] Muller D Karle A Meissburger B Hofig I Stork R Kontermann RE Improved pharmacokinetics of recombinant bispecific antibody molecules by fusion to human serum albumin. J Biol Chem (2007) 282(17):12650–60. 10.1074/jbc.M700820200 17347147

[53] Veronese FM Mero A. The impact of PEGylation on biological therapies. BioDrugs (2008) 22(5):315–29. 10.2165/00063030-200822050-00004 18778113

[54] Bell A Wang ZJ Arbabi-Ghahroudi M Chang TA Durocher Y Trojahn U Differential tumor-targeting abilities of three single-domain antibody formats. Cancer Lett (2010) 289(1):81–90. 10.1016/j.canlet.2009.08.003 19716651

[55] Heredia A Latinovic OS Barbault F de Leeuw EP. A novel small-molecule inhibitor of HIV-1 entry. Drug Des Devel Ther (2015) 9:5469–78. 10.2147/DDDT.S89338 26491257 PMC4598220

[56] Qiu LP Chen L Chen KP. Antihepatitis B therapy: a review of current medications and novel small molecule inhibitors. Fundam Clin Pharmacol (2014) 28(4):364–81. 10.1111/fcp.12053 24118072

[57] Meanwell NA Philip S Portoghese medicinal chemistry lectureship. curing hepatitis C virus infection with direct-acting antiviral agents: the arc of a medicinal chemistry triumph. J Med Chem (2016) 59(16):7311–51. 10.1021/acs.jmedchem.6b00915 27501244

[58] Lee KK Pessi A Gui L Santoprete A Talekar A Moscona A Capturing a fusion intermediate of influenza hemagglutinin with a cholesterol-conjugated peptide, a new antiviral strategy for influenza virus. J Biol Chem (2011) 286(49):42141–9. 10.1074/jbc.M111.254243 21994935 PMC3234914

[59] Flexner C Saag M. The antiretroviral drug pipeline: prospects and implications for future treatment research. Curr Opin HIV AIDS (2013) 8(6):572–8. 10.1097/COH.0000000000000011 24100879

[60] Vincke C Loris R Saerens D Martinez-Rodriguez S Muyldermans S Conrath K. General strategy to humanize a camelid single-domain antibody and identification of a universal humanized nanobody scaffold. J Biol Chem (2009) 284(5):3273–84. 10.1074/jbc.M806889200 19010777

[61] Plemper RK. Cell entry of enveloped viruses. Curr Opin Virol (2011) 1(2):92–100. 10.1016/j.coviro.2011.06.002 21927634 PMC3171968

[62] Tan M Hegde RS Jiang X. The P domain of norovirus capsid protein forms dimer and binds to histo-blood group antigen receptors. J Virol (2004) 78(12):6233–42. 10.1128/JVI.78.12.6233-6242.2004 15163716 PMC416535

[63] Klein JS Gnanapragasam PN Galimidi RP Foglesong CP West AP Jr Bjorkman PJ. Examination of the contributions of size and avidity to the neutralization mechanisms of the anti-HIV antibodies b12 and 4E10. Proc Natl Acad Sci U S A (2009) 106(18):7385–90. 10.1073/pnas.0811427106 19372381 PMC2678646

[64] Ouyang Y Yin Q Li W Li Z Kong D Wu Y Escape from humoral immunity is associated with treatment failure in HIV-1-infected patients receiving long-term antiretroviral therapy. Sci Rep (2017) 7(1):6222. 10.1038/s41598-017-05594-5 28740221 PMC5524822

[65] Worn A der Maur AA Escher D Honegger A Barberis A Pluckthun A. Correlation between in vitro stability and in vivo performance of anti-GCN4 intrabodies as cytoplasmic inhibitors. J Biol Chem (2000) 275(4):2795–803. 10.1074/Jbc.275.4.2795 10644744

[66] Xiang SH Doka N Choudhary RK Sodroski J Robinson JE. Characterization of CD4-induced epitopes on the HIV type 1 gp120 envelope glycoprotein recognized by neutralizing human monoclonal antibodies. AIDS Res Hum Retroviruses (2002) 18(16):1207–17. 10.1089/08892220260387959 12487827

[67] Wu X Yang ZY Li Y Hogerkorp CM Schief WR Seaman MS Rational design of envelope identifies broadly neutralizing human monoclonal antibodies to HIV-1. Science (2010) 329(5993):856–61. 10.1126/science.1187659 20616233 PMC2965066

[68] Walker LM Huber M Doores KJ Falkowska E Pejchal R Julien JP Broad neutralization coverage of HIV by multiple highly potent antibodies. Nature (2011) 477(7365):466–70. 10.1038/nature10373 21849977 PMC3393110

[69] Scheid JF Mouquet H Ueberheide B Diskin R Klein F Oliveira TY Sequence and structural convergence of broad and potent HIV antibodies that mimic CD4 binding. Science (2011) 333(6049):1633–7. 10.1126/science.1207227 21764753 PMC3351836

[70] Wyatt R Kwong PD Desjardins E Sweet RW Robinson J Hendrickson WA The antigenic structure of the HIV gp120 envelope glycoprotein. Nature (1998) 393(6686):705–11. 10.1038/31514 9641684

[71] Kwong PD Wyatt R Robinson J Sweet RW Sodroski J Hendrickson WA. Structure of an HIV gp120 envelope glycoprotein in complex with the CD4 receptor and a neutralizing human antibody. Nature (1998) 393(6686):648–59. 10.1038/31405 9641677 PMC5629912

[72] Berkower I Patel C Ni Y Virnik K Xiang Z Spadaccini A. Targeted deletion in the beta20-beta21 loop of HIV envelope glycoprotein gp120 exposes the CD4 binding site for antibody binding. Virology (2008) 377(2):330–8. 10.1016/j.virol.2008.03.040 18519142

[73] Labrijn AF Poignard P Raja A Zwick MB Delgado K Franti M Access of antibody molecules to the conserved coreceptor binding site on glycoprotein gp120 is sterically restricted on primary human immunodeficiency virus type 1. J Virol (2003) 77(19):10557–65. 10.1128/JVI.77.19.10557-10565.2003 12970440 PMC228502

[74] Frey G Peng H Rits-Volloch S Morelli M Cheng Y Chen B. A fusion-intermediate state of HIV-1 gp41 targeted by broadly neutralizing antibodies. Proc Natl Acad Sci U S A (2008) 105(10):3739–44. 10.1073/pnas.0800255105 18322015 PMC2268799

[75] Acharya P Luongo TS Georgiev IS Matz J Schmidt SD Louder MK Heavy chain-only IgG2b llama antibody effects near-pan HIV-1 neutralization by recognizing a CD4-induced epitope that includes elements of coreceptor- and CD4-binding sites. J Virol (2013) 87(18):10173–81. 10.1128/JVI.01332-13 23843638 PMC3753989

[76] Liu L Wang W Matz J Ye C Bracq L Delon J The glycosylphosphatidylinositol-anchored variable region of llama heavy chain-only antibody JM4 efficiently blocks both cell-free and T cell-T cell transmission of human immunodeficiency virus type 1. J Virol (2016) 90(23):10642–59. 10.1128/JVI.01559-16 27654286 PMC5110191

[77] McCoy LE Groppelli E Blanchetot C de Haard H Verrips T Rutten L Neutralisation of HIV-1 cell-cell spread by human and llama antibodies. Retrovirology (2014) 11:83. 10.1186/s12977-014-0083-y 25700025 PMC4189184

[78] Huang J Ofek G Laub L Louder MK Doria-Rose NA Longo NS Broad and potent neutralization of HIV-1 by a gp41-specific human antibody. Nature (2012) 491(7424):406–12. 10.1038/nature11544 23151583 PMC4854285

[79] Qiao ZS Kim M Reinhold B Montefiori D Wang JH Reinherz EL. Design, expression, and immunogenicity of a soluble HIV trimeric envelope fragment adopting a prefusion gp41 configuration. J Biol Chem (2005) 280(24):23138–46. 10.1074/jbc.M414515200 15833740

[80] Zhou M Kostoula I Brill B Panou E Sakarellos-Daitsiotis M Dietrich U. Prime boost vaccination approaches with different conjugates of a new HIV-1 gp41 epitope encompassing the membrane proximal external region induce neutralizing antibodies in mice. Vaccine (2012) 30(11):1911–6. 10.1016/j.vaccine.2012.01.026 22269872

[81] Ofek G Tang M Sambor A Katinger H Mascola JR Wyatt R Structure and mechanistic analysis of the anti-human immunodeficiency virus type 1 antibody 2F5 in complex with its gp41 epitope. J Virol (2004) 78(19):10724–37. 10.1128/JVI.78.19.10724-10737.2004 15367639 PMC516390

[82] Dimitrov DS Engineered CH2 domains (nanoantibodies). MAbs (2009) 1(1):26–8. 10.4161/mabs.1.1.7480 20046570 PMC2715188

[83] Bouchet J Basmaciogullari SE Chrobak P Stolp B Bouchard N Fackler OT Inhibition of the Nef regulatory protein of HIV-1 by a single-domain antibody. Blood (2011) 117(13):3559–68. 10.1182/blood-2010-07-296749 21292773

[84] Vercruysse T Pardon E Vanstreels E Steyaert J Daelemans D. An intrabody based on a llama single-domain antibody targeting the N-terminal alpha-helical multimerization domain of HIV-1 rev prevents viral production. J Biol Chem (2010) 285(28):21768–80. 10.1074/jbc.M110.112490 20406803 PMC2898381

[85] Bouchet J Herate C Guenzel CA Verollet C Jarviluoma A Mazzolini J Single-domain antibody-SH3 fusions for efficient neutralization of HIV-1 Nef functions. J Virol (2012) 86(9):4856–67. 10.1128/JVI.06329-11 22345475 PMC3347381

[86] Hause BM Collin EA Liu R Huang B Sheng Z Lu W Characterization of a novel influenza virus in cattle and Swine: proposal for a new genus in the Orthomyxoviridae family. MBio (2014) 5(2):e31–14. 10.1128/mBio.00031-14 24595369 PMC3958797

[87] Gao R Cao B Hu Y Feng Z Wang D Hu W Human infection with a novel avian-origin influenza A (H7N9) virus. N Engl J Med (2013) 368(20):1888–97. 10.1056/NEJMoa1304459 23577628

[88] Tscherne DM García-Sastre A. Virulence determinants of pandemic influenza viruses. J Clin Invest (2011) 121(1):6–13. 10.1172/jci44947 21206092 PMC3007163

[89] Basu A Antanasijevic A Wang M Li B Mills DM Ames JA New small molecule entry inhibitors targeting hemagglutinin-mediated influenza a virus fusion. J Virol (2014) 88(3):1447–60. 10.1128/JVI.01225-13 24198411 PMC3911584

[90] De Clercq E. Antiviral agents active against influenza A viruses. Nat Rev Drug Discov (2006) 5(12):1015–25. 10.1038/nrd2175 17139286 PMC7097821

[91] Tutykhina IL Sedova ES Gribova IY Ivanova TI Vasilev LA Rutovskaya MV Passive immunization with a recombinant adenovirus expressing an HA (H5)-specific single-domain antibody protects mice from lethal influenza infection. Antiviral Res (2013) 97(3):318–28. 10.1016/j.antiviral.2012.12.021 23274786

[92] Pinto LH Holsinger LJ Lamb RA Influenza-virus M2 protein has ion channel activity. Cell (1992) 69(3):517–28. 10.1016/0092-8674(92)90452-I 1374685

[93] Palese P Shaw ML Orthomyxoviridae: the viruses and their replication. In: Ed Knipe DM Howley PM Griffin DE Lamb RA Martin MA Roizman B, editors. Fields Virology. Philadelphia, PA: Lippincott Williams & Wilkins (2007) 1647–89.

[94] Nair H Nokes DJ Gessner BD Dherani M Madhi SA Singleton RJ Global burden of acute lower respiratory infections due to respiratory syncytial virus in young children: a systematic review and meta-analysis. Lancet (2010) 375(9725):1545–55. 10.1016/S0140-6736(10)60206-1 20399493 PMC2864404

[95] Battles MB Langedijk JP Furmanova-Hollenstein P Chaiwatpongsakorn S Costello HM Kwanten L Molecular mechanism of respiratory syncytial virus fusion inhibitors. Nat Chem Biol (2016) 12(2):87–93. 10.1038/nchembio.1982 26641933 PMC4731865

[96] Johnson S Oliver C Prince GA Hemming VG Pfarr DS Wang SC Development of a humanized monoclonal antibody (MEDI-493) with potent in vitro and in vivo activity against respiratory syncytial virus. J Infect Dis (1997) 176(5):1215–24. 10.1086/514115 9359721

[97] Group TI-RS Palivizumab, a humanized respiratory syncytial virus monoclonal antibody, reduces hospitalization from respiratory syncytial virus infection in high-risk infants. Pediatrics (1998) 102(3):531–7. 10.1542/peds.102.3.531 9724660

[98] Corti D Bianchi S Vanzetta F Minola A Perez L Agatic G Cross-neutralization of four paramyxoviruses by a human monoclonal antibody. Nature (2013) 501(7467):439–43. 10.1038/nature12442 23955151

[99] Zhao M Zheng ZZ Chen M Modjarrad K Zhang W Zhan LT Discovery of a prefusion respiratory syncytial virus F-specific monoclonal antibody that provides greater in vivo protection than the murine precursor of palivizumab. J Virol (2017) 91(15):e176–117. 10.1128/JVI.00176-17 28539438 PMC5651723

[100] Capella C Chaiwatpongsakorn S Gorrell E Risch ZA Ye F Mertz SE Prefusion F, postfusion F, G antibodies and disease severity in infants and young children with acute respiratory syncytial virus infection. J Infect Dis (2017). 10.1093/infdis/jix489 29029312 PMC5853469

[101] Gentile I Maraolo AE Buonomo AR Zappulo E Borgia G. The discovery of sofosbuvir: a revolution for therapy of chronic hepatitis C. Expert Opin Drug Discov (2015) 10(12):1363–77. 10.1517/17460441.2015.1094051 26563720

[102] Khan AG Whidby J Miller MT Scarborough H Zatorski AV Cygan A Structure of the core ectodomain of the hepatitis C virus envelope glycoprotein 2. Nature (2014) 509(7500):381–4. 10.1038/nature13117 24553139 PMC4126800

[103] Law M Maruyama T Lewis J Giang E Tarr AW Stamataki Z Broadly neutralizing antibodies protect against hepatitis C virus quasispecies challenge. Nat Med (2008) 14(1):25–7. 10.1038/nm1698 18064037

[104] Morin TJ Broering TJ Leav BA Blair BM Rowley KJ Boucher EN Human monoclonal antibody HCV1 effectively prevents and treats HCV infection in chimpanzees. PLoS Pathog (2012) 8(8):e1002895. 10.1371/journal.ppat.1002895 22952447 PMC3431327

[105] Chung RT Gordon FD Curry MP Schiano TD Emre S Corey K Human monoclonal antibody MBL-HCV1 delays HCV viral rebound following liver transplantation: a randomized controlled study. Am J Transplant (2013) 13(4):1047–54. 10.1111/ajt.12083 23356386 PMC3618536

[106] Looker KJ Gamett GP Schmid GP. An estimate of the global prevalence and incidence of herpes simplex virus type 2 infection. Bull World Health Organ (2008) 86(10):805–12. 10.2471/BLT.07.046128 18949218 PMC2649511

[107] Holmberg SD Stewart JA Gerber AR Byers RH Lee FK Omalley PM Prior herpes-simplex virus type-2 infection as a risk factor for HIV infection. J Am Med Assoc (1988) 259(7):1048–50. 10.1001/Jama.259.7.1048 2828700

[108] Atanasiu D Saw WT Cohen GH Eisenberg RJ. Cascade of events governing cell-cell fusion induced by herpes simplex virus glycoproteins gD, gH/gL, and gB. J Virol (2010) 84(23):12292–9. 10.1128/JVI.01700-10 20861251 PMC2976417

[109] Nicola AV Ponce de Leon M Xu R Hou W Whitbeck JC Krummenacher C Monoclonal antibodies to distinct sites on herpes simplex virus (HSV) glycoprotein D block HSV binding to HVEM. J Virol (1998) 72(5):3595–601. 9557640 10.1128/jvi.72.5.3595-3601.1998 PMC109580

[110] Pant N Hultberg A Zhao Y Svensson L Pan-Hammarstrom Q Johansen K Lactobacilli expressing variable domain of llama heavy-chain antibody fragments (lactobodies) confer protection against rotavirus-induced diarrhea. J Infect Dis (2006) 194(11):1580–8. 10.1086/508747 17083044

[111] Pant N Marcotte H Hermans P Bezemer S Frenken L Johansen K Lactobacilli producing bispecific llama-derived anti-rotavirus proteins in vivo for rotavirus-induced diarrhea. Future Microbiol (2011) 6(5):583–93. 10.2217/fmb.11.32 21585264

[112] Sarker SA Jakel M Sultana S Alam NH Bardhan PK Chisti MJ Anti-rotavirus protein reduces stool output in infants with diarrhea: a randomized placebo-controlled trial. Gastroenterology (2013) 145(4):740–8.e8. 10.1053/j.gastro.2013.06.053 23831050

[113] Hansman GS Natori K Shirato-Horikoshi H Ogawa S Oka T Katayama K Genetic and antigenic diversity among noroviruses. J Gen Virol (2006) 87(Pt 4):909–19. 10.1099/vir.0.81532-0 16528040

[114] Hansman GS Taylor DW McLellan JS Smith TJ Georgiev I Tame JR Structural basis for broad detection of genogroup II noroviruses by a monoclonal antibody that binds to a site occluded in the viral particle. J Virol (2012) 86(7):3635–46. 10.1128/JVI.06868-11 22278249 PMC3302548

[115] Shiota T Okame M Takanashi S Khamrin P Takagi M Satou K Characterization of a broadly reactive monoclonal antibody against norovirus genogroups I and II: recognition of a novel conformational epitope. J Virol (2007) 81(22):12298–306. 10.1128/JVI.00891-07 17855545 PMC2168978

[116] Ryckaert S Pardon E Steyaert J Callewaert N. Isolation of antigen-binding camelid heavy chain antibody fragments (nanobodies) from an immune library displayed on the surface of Pichia pastoris. J Biotechnol (2010) 145(2):93–8. 10.1016/j.jbiotec.2009.10.010 19861136

[117] Ying T Chen W Feng Y Wang Y Gong R Dimitrov DS. Engineered soluble monomeric IgG1 CH3 domain: generation, mechanisms of function, and implications for design of biological therapeutics. J Biol Chem (2013) 288(35):25154–64. 10.1074/jbc.M113.484154 23867459 PMC3757179

[118] Ying T Ju TW Wang Y Prabakaran P Dimitrov DS. Interactions of IgG1 CH2 and CH3 domains with FcRn. Front Immunol (2014) 5:146. 10.3389/fimmu.2014.00146 24765095 PMC3980117

[119] Ying T Wang Y Feng Y Prabakaran P Gong R Wang L Engineered antibody domains with significantly increased transcytosis and half-life in macaques mediated by FcRn. MAbs (2015) 7(5):922–30. 10.1080/19420862.2015.1067353 26179052 PMC4622838

[120] Ying T Feng Y Wang Y Chen W Dimitrov DS. Monomeric IgG1 Fc molecules displaying unique Fc receptor interactions that are exploitable to treat inflammation-mediated diseases. MAbs (2014) 6(5):1201–10. 10.4161/mabs.29835 25517305 PMC4622432

[121] Wang L Ying T. New directions for half-life extension of protein therapeutics: the rise of antibody Fc domains and fragments. Curr Pharm Biotechnol (2016) 17(15):1348–52. 10.2174/1389201017666160823144032 27552847

[122] Jespers L Schon O James LC Veprintsev D Winter G Crystal structure of HEL4, a soluble, refoldable human VH single domain with a germ-line scaffold. J Mol Biol (2004) 337(4):893–903. 10.1016/j.jmb.2004.02.013 15033359

[123] Dudgeon K Rouet R Kokmeijer I Schofield P Stolp J Langley D General strategy for the generation of human antibody variable domains with increased aggregation resistance. Proc Natl Acad Sci U S A (2012) 109(27):10879–84. 10.1073/pnas.1202866109 22745168 PMC3390889

